# Achieving broad absorption band and high incident angles by stochastically-distributed oblique-flat-sheet metamaterial perfect absorbers

**DOI:** 10.1038/s41598-021-98077-7

**Published:** 2021-09-21

**Authors:** Cheng-Yu Lu, Chin-Chien Chung, Ta-Jen Yen, Tsung-Yu Huang

**Affiliations:** 1 Department of Materials Engineering, Ming Chi University of Technology, New Taipei, 24301 Taiwan, ROC; 2 Department of Materials Science and Engineering, National Tsing Hua University, Hsinchu, 30013 Taiwan, ROC

**Keywords:** Metamaterials, Applied optics, Nanophotonics and plasmonics

## Abstract

In this work, we integrated a periodic seed layer and oblique deposition method to fabricate a stochastically-distributed oblique-flat-sheet metamaterial perfect absorber (MPA). Such design could increase its absorption bandwidth and tolerance to high angle-incidence due to the fact that various oblique flat sheets offer different resonance conditions while even a single oblique flat sheet could provide different optical paths for resonance. On the other hand, a seed layer could reduce uncertainty regarding to direct oblique deposition and provide abilities to manipulate the bandwidth of the MPA. We also setup a simulation model in the aids of Visual Basic Application and examined the absorption behavior of the MPA under TM and TE oblique incidence that could achieve high absorbance under 80° and 60° incidence, respectively. Finally, in measurement, the fabricated sample owns 65% absorbance within 80–250 THz and over 90% absorbance within 250–320 THz at x-polarization normal incidence; as for the y-polarization normal incidence, we could achieve overall 70% absorbance within 80–300 THz. The measured results reveal similar tendency compared to the simulated ones.

## Introduction

Within these two decades, the emergence of metamaterials^1–4^ and metasurfaces^5–7^ has changed the design guideline of an optical device. Furthermore, ever since in 2008 Landy et al*.*^8^ proposed metamaterial perfect absorbers (MPAs) with a unity absorption and a subwavelength thickness, this concept soon captured researchers’ attention. MPAs were founded on patterned metal/dielectric/metal three layered structures. The structure could provide electric and magnetic responses, simultaneously, to match free space impedance. On the other hand, the bottom cut wires could block all transmitted light at the operating frequency. Till now, several perfect absorbers with different functionalities were proposed, for example, MPAs at microwave^9,10^, infrared^11,12^ and visible ranges^13^, MPAs with dual^14,15^, triple^16^ and multiple absorption bands^17^ and active MPAs with vanadium dioxide^18^ as optical switches. Moreover the emergence of strong demand regarding to broad bandwidth and large incident angles in the fields of bolometers^19,20^, photodetection^21^, stealthy technology^22^ and energy harvesting^13,23^, have stimulated the research enthusiasm toward MPAs with broad bandwidth and large incidence angles. Different design concepts are proposed such as coplanar MPAs with similar but different patterns^24,25^, stacked MPAs with various patterns stacked along their heights^26–29^, MPAs with lumped elements^30,31^, and MPAs with randomly distributed patterns^32^. The effective bandwidth could be boosted by all of these methods^33,34^; yet, there still exist some insufficiencies, for example, coupling among resonators for co-planar MPAs, thicker thicknesses and fabrication difficulty for stacked MPAs, microwave regime only for lumped-element based MPAs and finally unpredictable behavior of MPAs with randomly distributed patterns. To solve the abovementioned issues, we proposed oblique-flat-sheet (OFS) MPAs that integrated a periodic seed layer and oblique deposition method together. Such design could increase its absorption bandwidth and tolerance to high angle-incidence due to the fact that a single oblique flat sheet (SOFS) could provide different optical paths for both bandwidth and incident angle enhancement and during oblique deposition, the fabricated sample revealed multiple oblique flat sheets (MOFS) that offer different resonance conditions and further boost the allowed bandwidth and incident angles. Also, a seed layer could reduce uncertainty regarding to complete randomly distributed patterns and provide abilities to manipulate the bandwidth of the MPA. Finally, the fabrication procedure is identical to the ones of conventional MPAs; the only difference is that our proposed OFS MPAs tilted an angle with respect to sample holder’s normal during deposition; thus, we believe that stochastically-distributed oblique-flat-sheet MPAs could outperform the abovementioned MPAs without additional fabrication burdens.

**Numerical simulations**

**Figure 1:**
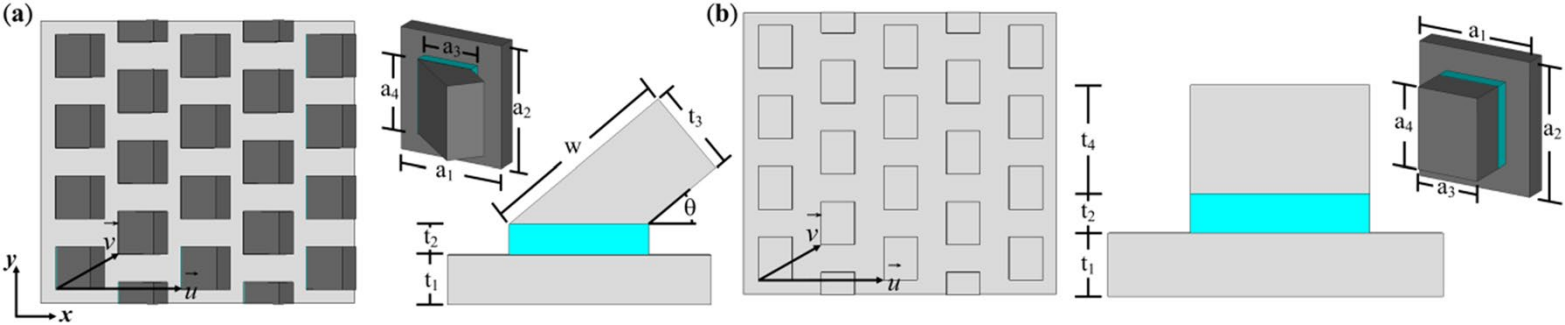
Schemes of (**a**) a single-oblique-flat-sheet metamaterial perfect absorber (SOFS MPA) and (**b**) a planar MPA. Detailed dimensions are a_1_ = 740 nm, a_2_ = 834 nm, a_3_ = 396 nm, a_4_ = 508 nm, t_1_ = 150 nm, t_2_ = 80 nm, t_3_ = 253 nm, w = 550 nm, θ = 40°, t_4_ = 240 nm, $\mathop{u}\limits^{\rightharpoonup} = 2a_{1} \vec{x}$ and $\mathop{v}\limits^{\rightharpoonup} = a_{1} \vec{x} + 0.5 \times a_{2} \vec{y}$.

To verify our proposed OFS MPAs, first, we want to confirm that compared to conventional planar MPAs, an SOFS MPA itself could provide different optical paths beneath the sheet, thus enabling multiple absorption bands. To validate such expectation, we designed an SOFS MPA as shown in Fig. 1a with aluminum for metal of the top and bottom layers and MgF_2_ (ε = 2.0 and tan δ = 0.001) for the spacer. It is worth mentioning that the low loss metal and dielectric are chosen because we want to emphasize on the resonance absorption from our proposed design instead of material absorption which is intrinsic to material properties and difficult to manipulate. Several dimensional parameters are restricted detailed below. First of all, θ i.e., the angle between the oblique flat sheet and the substrate depends on the titled angles with respect to sample holder’s normal and also deposited metal. On the other hand, in order to avoid residual flat sheets growing on the gaps of the seed layers, the translational vectors of the array are $\mathop{u}\limits^{\rightharpoonup}$ and $\mathop{v}\limits^{\rightharpoonup}$ whereas $\mathop{u}\limits^{\rightharpoonup} = 2a_{1} \vec{x}$ and $\mathop{v}\limits^{\rightharpoonup} = a_{1} \vec{x} + 0.5 \times a_{2} \vec{y}$ as shown in Fig. 1a for a periodic array. Through this design, we could not only prevent additional sheets on the gaps but also suppress higher-order reflection^35,36^. Finally, the thickness and dimension of a seed layer could be predicted by the formula $\mathrm{s}<\mathrm{tan\alpha }\times \mathrm{h}$^37^ whereas s is the coverage range for the shadow effect, α the angle for the incident flux with respect to the substrate’s normal and h the thickness of the seed layer. In experiments, α is 86° for 40° inclination angle and a coverage range of 1144 nm. Therefore, the corresponding dimensions are a_1_ = 740 nm, a_2_ = 834 nm, a_3_ = 396 nm, a_4_ = 508 nm, t_1_ = 150 nm, t_2_ = 80 nm, t_3_ = 253 nm, w = 550 nm, and θ = 40°. Besides, a conventional planar MPA with the same seed layer is also simulated for comparison. After optimization, the metal thickness of the upper rectangle is 240 nm for the planar MPA as illustrated in Fig. 1b. In simulation, we employed finite integration method with unit cell boundary conditions for the SOFS and planar MPAs. To obtain absorbance, the formula for energy conservation is used, i.e., A = 1 – R − T where A is absorbance, R reflectance and T transmittance. Note that once we match the impedance of the MPAs with free space’s, one can reduce R to 0 while T is 0 since the continuous grounded metal could block all the transmitted wave. Here, we target our frequency range within 20–320 THz in consideration of experimental measurement.

**Figure 2:**
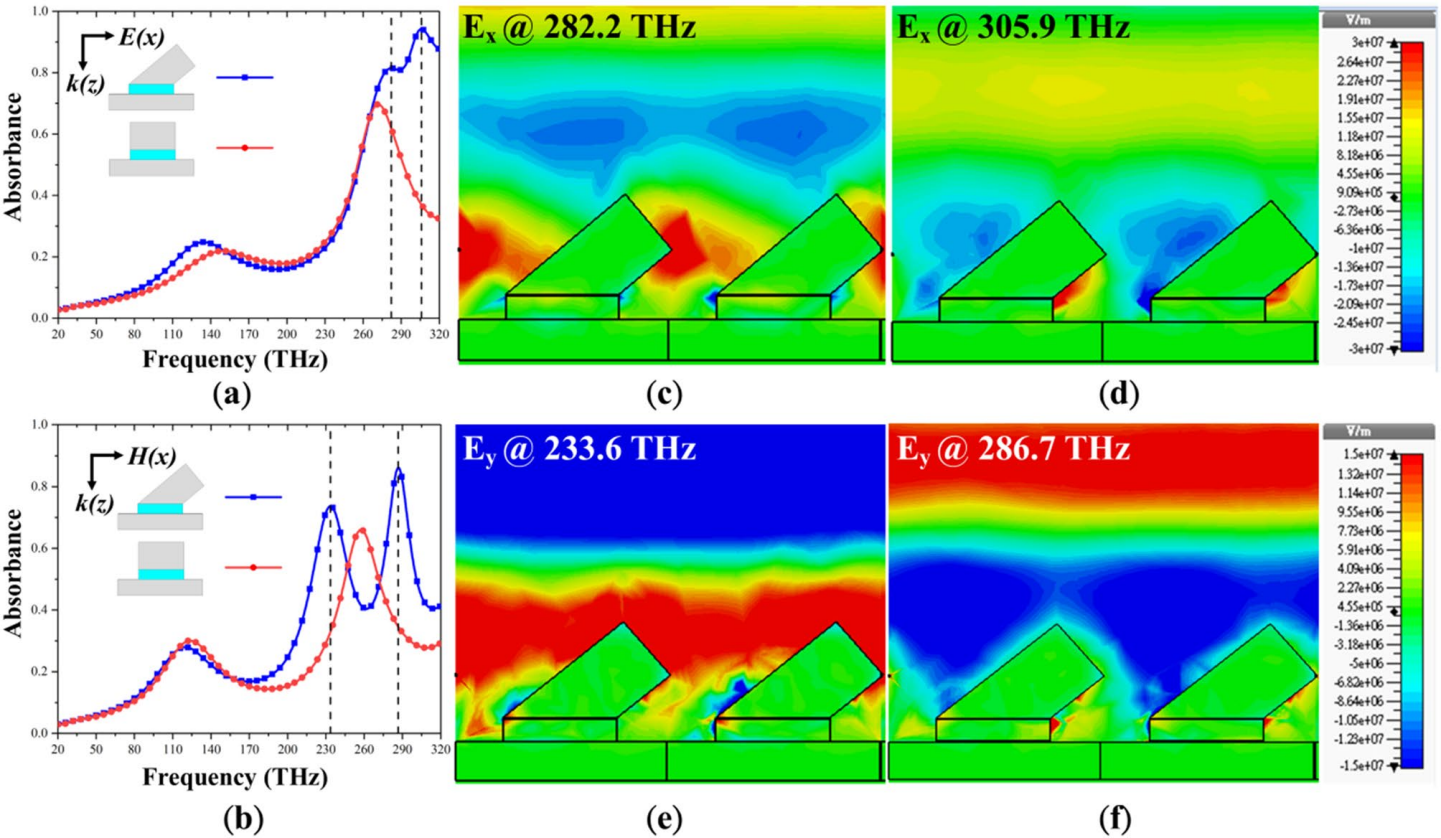
Absorbance of the SOFS and planar MPAs under normal incidence at (**a**) x- and (**b**) y-polarization, respectively. Absorbance of the SOFS MPA are larger than the ones of the planar MPA for both polarizations. Recorded field distributions of the SOFS MPA at the two absorbance peaks for (**c**,**d**) x- and (**e**,**f**) y-polarization. Field distributions indicate that an oblique flat sheet could provide different penetration routes (top edge and bottom side) for the incoming wave, thus providing a different phase change for resonance.

Figure 2a,b depicts absorbance spectra for the SOFS and planar MPAs at two different polarizations. At x-polarization, compared to the planar MPA with only one absorbance peak of 69.73% at 271.7 THz, there appear two profound peaks with absorbance of 81% and 94.22% at 282.2 and 305.9 THz, respectively. To further distinguish the differences of the two, we integrate the absorbance spectra within the frequency range. The integrated absorbance equal to 90.46 and 71.19 for the SOFS and planar MPAs, respectively; the integrated absorbance of the SOFS MPA is 27% better than the one of the planar MPA. As for the y-polarization, one absorbance peak of 66% at 257.6 THz for the planar MPA and two absorbance peaks of 73.7% and 86.12% at 233.6 and 286.7 THz for the SOFS MPA are observed. Also, the integrated absorbance equal to 92.28 and 70.88 for the SOFS and planar MPAs that is an enhancement of 30.19%. The corresponding field distributions are monitored to evidence the different optical paths at different peaks. For both x- and y-polarization, we could observe that at the lower frequency, the field concentrated at the top edges of the oblique flat sheet as shown in Fig. 2c,e; on the other hand, at the higher frequency, the field mainly focused on the bottom side of the oblique flat sheet as portrayed in Fig. 2d,f. Since the fields locate within air and the propagation route are different, the two different frequencies experience different optical phase differences. To sum up, these simulations and field distributions have validated our expectation that with a single oblique flat sheet as the upper resonator of the MPA, one can provide different optical phase differences, resulting in multiple absorbance peaks.

**Figure 3:**
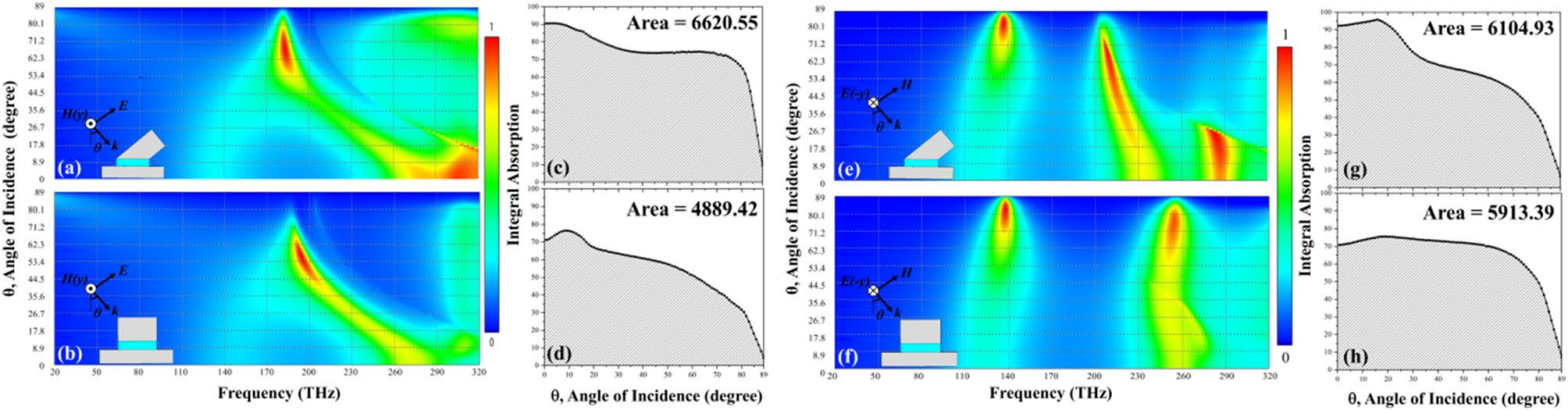
Absorbance diagram with respect to frequency and incident angles of the (**a**,**e**) SOFS and (**b**,**f**) planar MPAs for (**a**,**b**) TM and (**e**,**f**) TE modes. The integral absorbance plots with respect to the incident angle of the (**c**,**g**) SOFS and (**d**,**h**) planar MPAs for (**c**,**d**) TM and (**g**,**h**) TE modes. The integral areas of the SOFS and planar MPAs are 6620.55 and 4889.42 for TM mode and 6104.93 and 5913.93 for TE mode, respectively.

So far, we have demonstrated our proposed SOFS MPA could possess better absorption behavior under normal incidence compared to the planar counterpart and then we would like to examine its behavior under oblique angle incidence. Figure 3 illustrated the corresponding absorbance with incident angle from 0 to 89° and frequency from 20 to 320 THz. For the TM mode as shown in Fig. 3a,b for the SOFS and planar MPA, respectively, the additional peak for the SOFS MPA disappeared after 17.5° incidence. Other than this frequency range, the two show similar absorbance trend with respect to increasing incident angles that the absorbance gradually decreased till 50° and then increased again while the operating frequency red shifts from 280 to 180 THz. Next, we integrated the absorbance curve under frequency with respect to the incident angle to quantitatively compare the absorbance behavior of the two MPAs. The integrated absorbance for the SOFS MPAs are 6620.55 which is 35% better than 4889.42 of the planar MPA as shown in Fig. 3c,d, respectively. On the other hand, for the TE mode as plotted in Fig. 3e,f, the main difference between the SOFS and planar MPAs stems from the additional peak at the higher frequency range and lower incident angles. Again, this additional peak disappeared over 36° incident angle. Yet, the first absorbance peak of the SOFS MFA gradually increased with increasing incident angle from 18° to 80° which are much wider compared to the allowed angles from 60° to 82° of the planar MPA. Finally, as illustrated in Fig. 3g,h, the integrated absorbance with respect to incident angle of the SOFS MPA is larger than the counterpart of the planar MPA before 70° but then drop much quickly. Still, the integrated absorbance for the SOFS MPA are 6104.93 which is 3.2% better than 5913.39 of the planar MPA. From the abovementioned comparison, we could claim that the proposed SOFS MPA could widen the absorption band and also the allowed incident angles.

**Figure 4:**
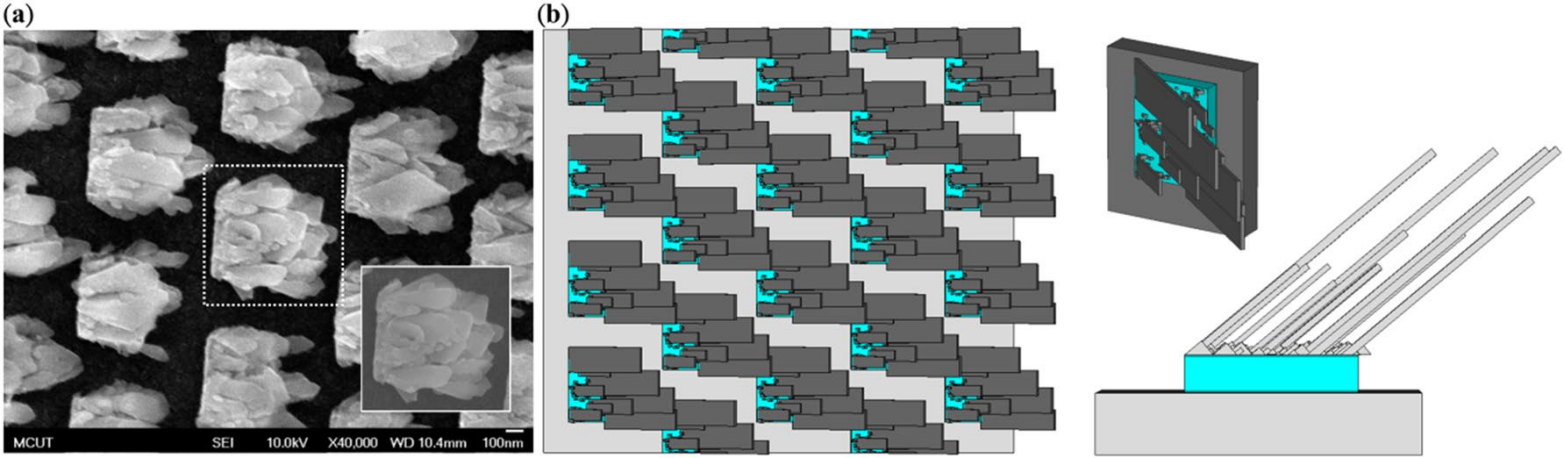
(**a**) SEM image of the fabricated multiple-oblique-flat-sheet (MOFS) MPA. (**b**) Constructed MOFS MPA in the aids of Visual Basic Application.

**Figure 5:**
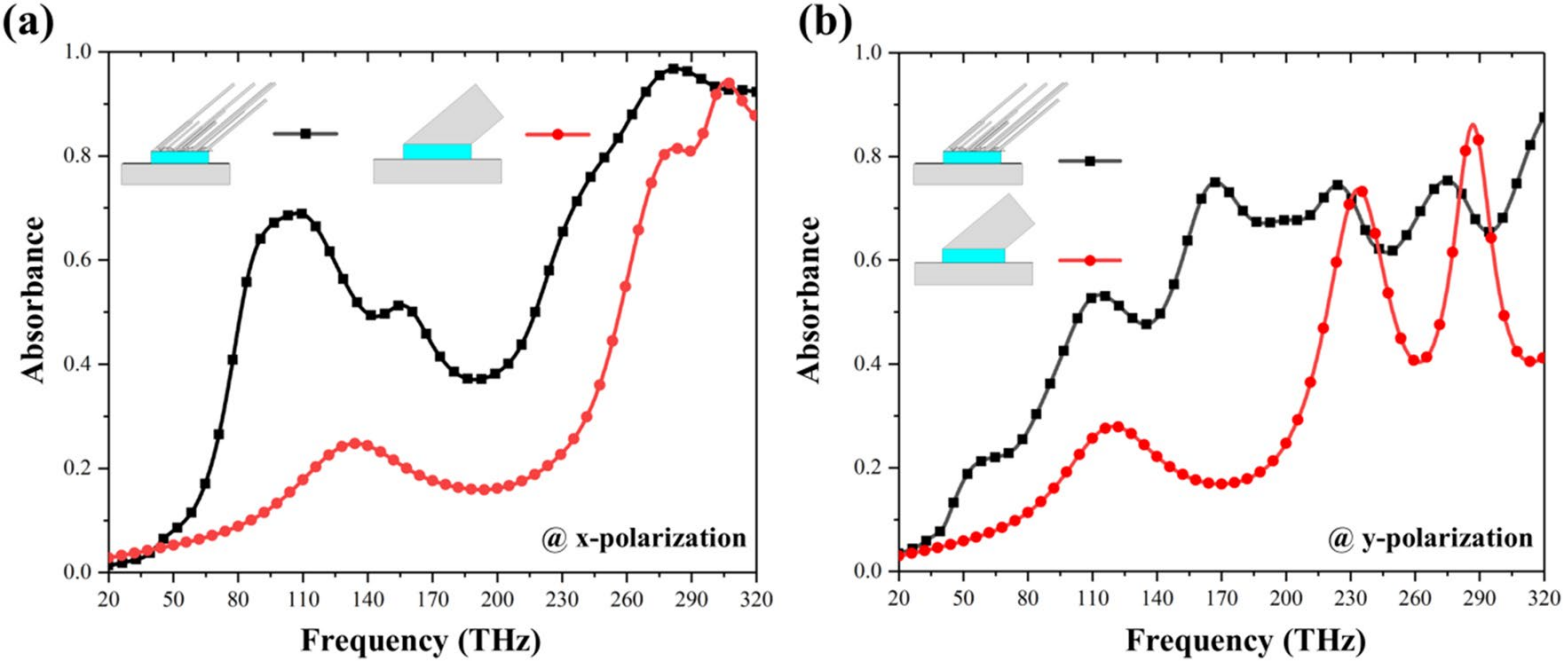
Absorbance of MOFS and the planar MPAs under normal incidence at (**a**) x- and (**b**) y-polarization, respectively. Absorbance are larger for MOFS MPA at both polarization, which could be attributed to different sizes and gap distance among the oblique flat sheets, thus resulting in different resonance conditions and coupling strength among resonances.

Although the SOFS MPA itself could provide a broadband absorption and wide incident angles, still, in experiments, instead of a single oblique flat sheet, we will obtain multiple oblique flat sheets with various dimensions randomly distributed on the seed layer. Also, we would like to boost the absorption band and incident angles further to a larger range. Thus, we employed Visual Basic Application (VBA) to construct a stochastically distributed MOFS MPA based on the SEM images of the fabricated sample as illustrated in Fig. 4. Construction procedure is detailed in “Methods”. Through this simulation, we can predict the absorption behavior of our proposed MOFS MPA and compare with the measurement results. Also, we could predict the behavior of oblique angle incidence since we are lack of facilities to measure absorption under oblique incidence condition. Figure 5 plots the absorbance spectra at the two different polarizations. The absorbance of the SOFS MPA is also included for comparison. From Fig. 5, the absorbance is larger at both polarizations compared to the ones of SOFS MPA and is attributed to the multiple oblique flat sheets with so many different dimensions and also different gap distances among the oblique flat sheets that contributed to different resonance conditions and coupling strength among resonances^32–34^.

**Figure 6:**
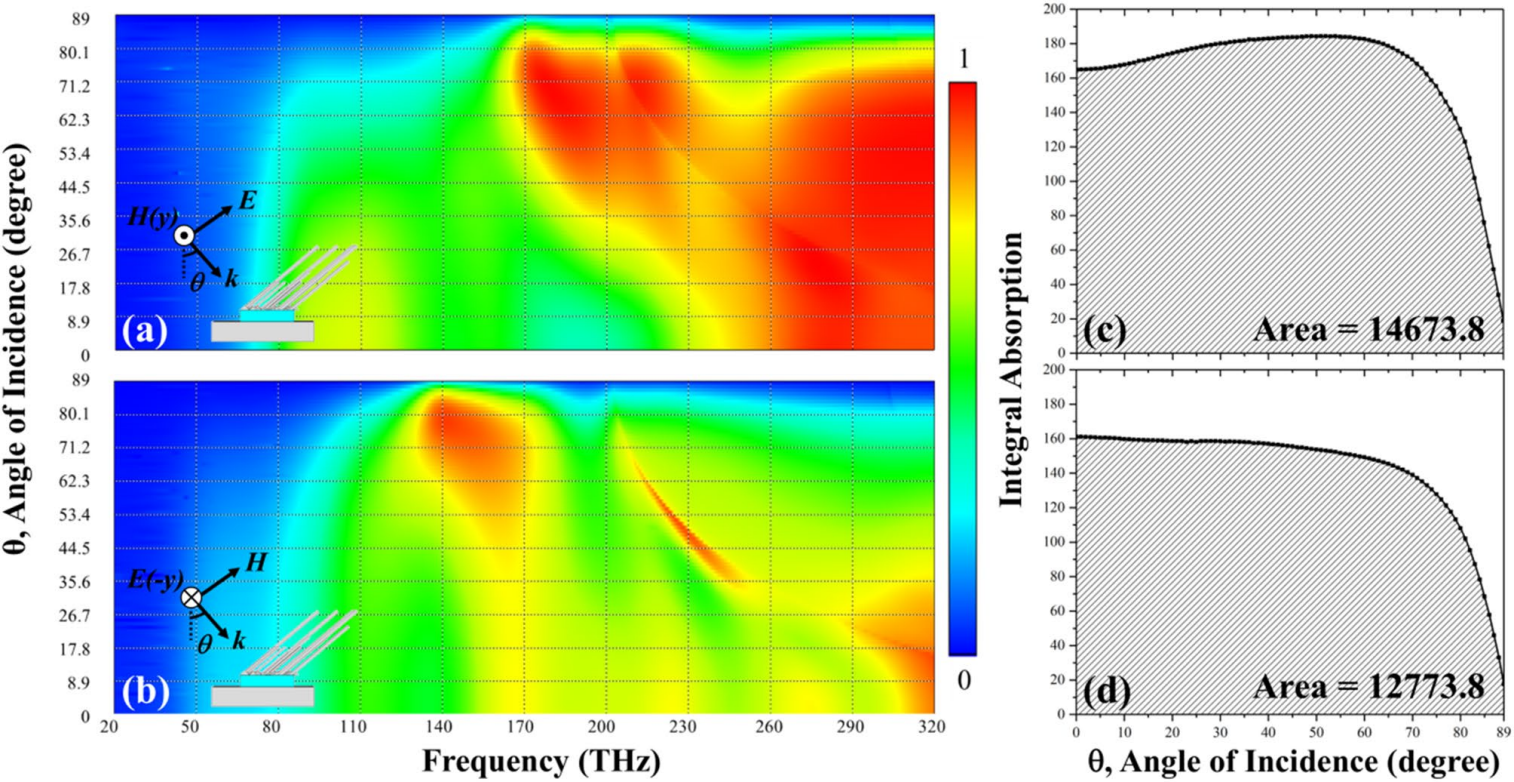
Absorbance diagram with respect to frequency and incident angles of SOFS MPAs for (**a**) TM and (**b**) TE modes. The integral absorbance plots with respect to the incident angle of SOFS MPAs for (**c**) TM and (**d**) TE modes. The integral areas of SOFS MPA are 14,673.8 and 12,773.8 for the TM and TE modes, respectively.

Regarding to the absorbance of the MOFS MPA under oblique incidence as shown in Fig. 6a,b for the TM and TE modes, respectively, first, in Fig. 6a, the absorbance drops with increasing incident angles at around 104 THz; after 50° incident angle, the absorbance became minor; on the other hand, the absorbance shows significant peaks before 80° incident angle with a red-shifted frequency from 320 to 170 THz with the increasing incident angle. As for the TE mode illustrated in Fig. 6b, overall absorbance is not quite high compared to the TM mode and the absorbance peak would gradually increase with red-shifted resonance frequency from 170 to 140 THz; at 320 THz, the peak gradually decreased till 25° and then increased again up to 72° with red-shifted resonance frequency also. Here, we conclude that when the incident E field is parallel to the MOFS, we could obtain higher absorbance enhancement compared to the normal one. Then, we conduct the integration of absorbance with respect to the frequency and incident angle. As shown in Fig. 6c,d, the integrated absorbance gradually increased with respect to the incident angle till 60° and then decreased. The absorbance reached to the value under normal incidence at 70° and started to drop much more quickly. The overall absorbance is 14,673.8 that is much larger than 6620.55 of the SOFS MPA and shows 122% enhancement. For TE mode, the absorbance kept almost constant till 70° and then dropped. Still, the overall absorbance is 12,773.8 compared to 6104.93 of the SOFS MPA and reveals a 109% boost. To sum up, we believe that with multiple oblique flat sheets, we can widen the absorption bandwidth under much wider incident angles.

## Fabrication and measurement

**Figure 7:**
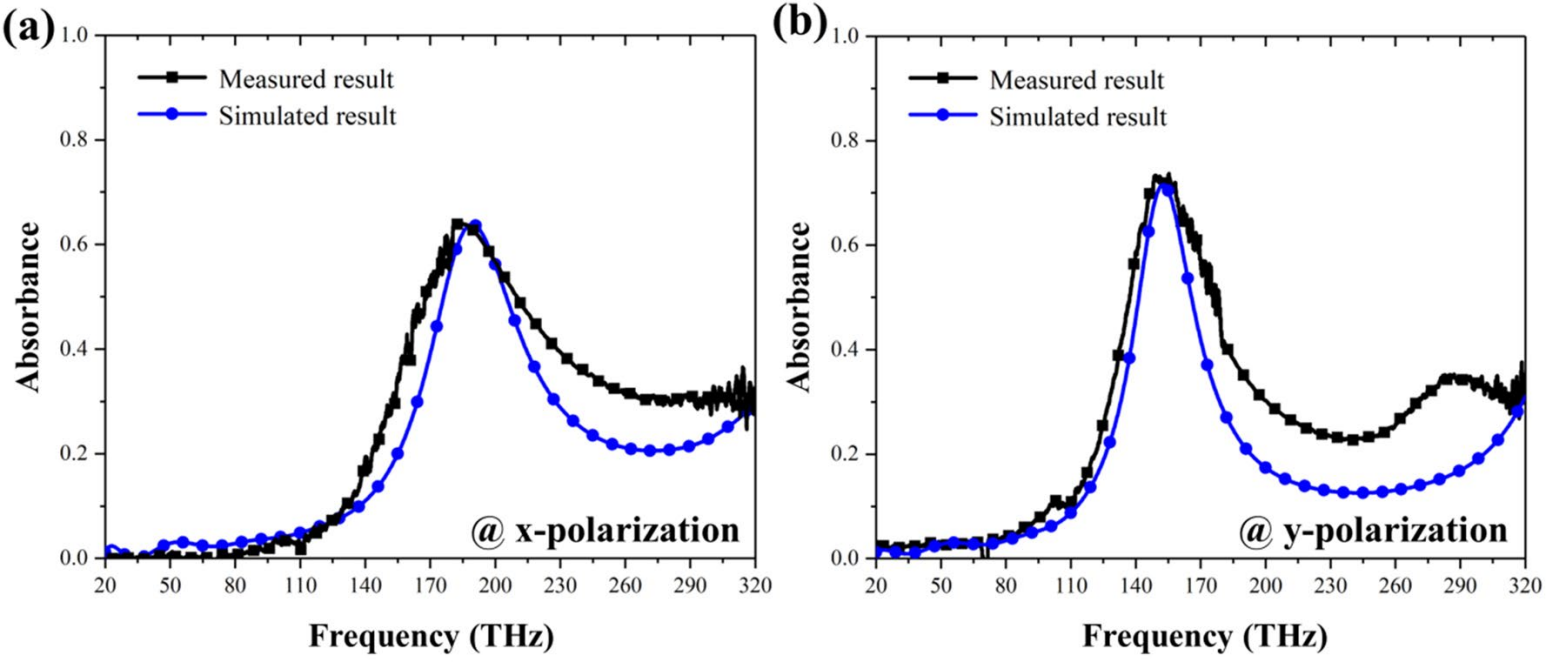
Measured absorbance of the planar MPA at (**a**) x- and (**b**) y-polarization. Measured absorbance agrees with the simulated ones well, establishing the correctness of material properties in simulation.

The success in simulation has encouraged us to continue the experimental measurement. To fabricate the planar and MOFS MPAs, we deposited a 150-nm-thick aluminum ground plane, conduct e-beam lithography procedure for a periodic MgF_2_ seed layer with a thickness of 80 nm and a periodicity of 740 and 834 nm in x and y directions, respectively. The as-fabricated sample was then fixed on the sample holder without and with a tilted angle of 86° with respect to the horizon and conducted normal and oblique angle deposition, respectively. Note that the difference between the simulated and fabricated planar MPA is its metal thickness (240 nm vs. 30 nm), and only the operating frequency shifts to lower one with a minor decrease of absorption. The sample was measured by µ-FTIR with two detectors (one for 20–179 THz and the other for 180–320 THz). As shown in Fig. 7a,b, the simulated and measured results of the planar MPA at the two polarizations agree each other well with the same resonance frequency and about 10% higher absorbance. Such difference could be attributed to the roughness of the thin film^38^. Also, through this measurement, we could confirm the material properties used in simulation should be correct.

**Figure 8:**
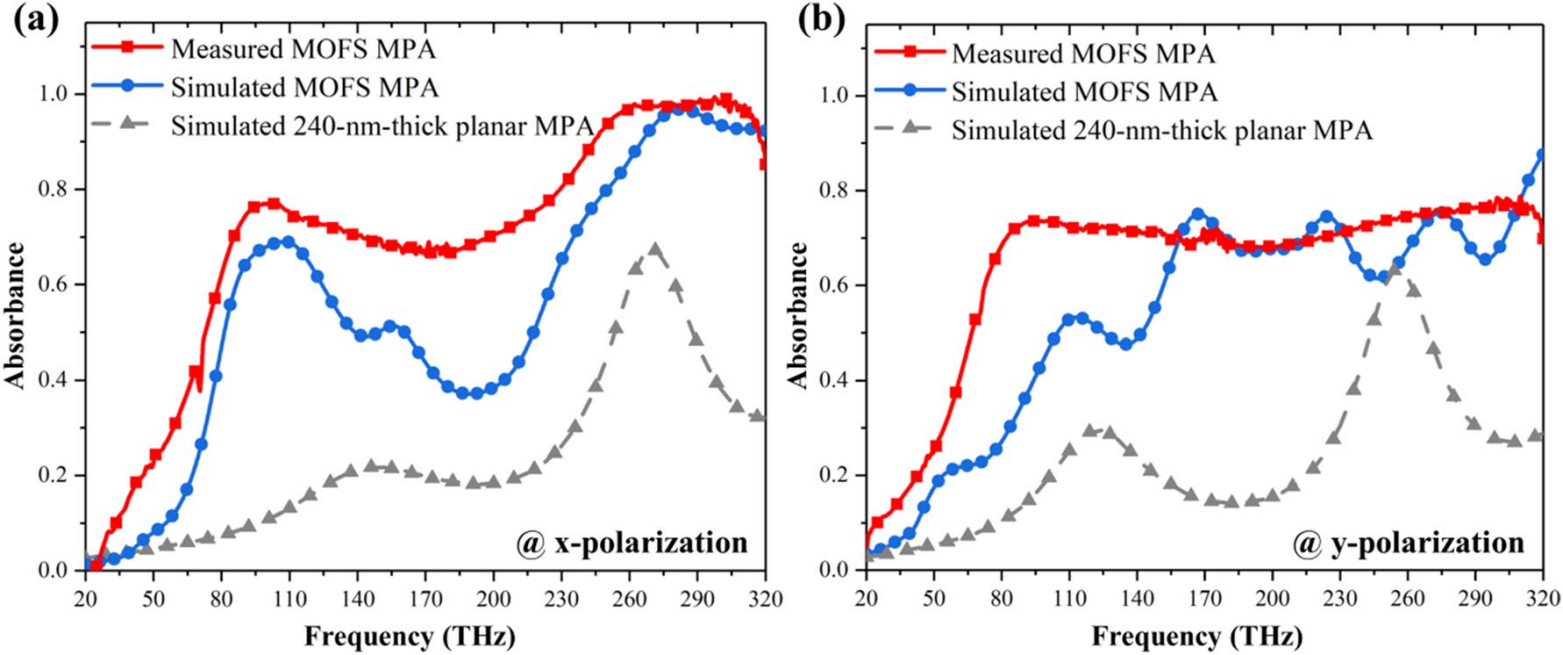
Absorbance of MOFS MPA for (**a**) x- and (**b**) y-polarization. Simulated absorbance of the planar MPA is also included for comparison. Measured and simulated absorbance of MOFS MPA follow the similar trend and outweigh the one of the planar MPA.

Figure 8 reveals the comparison of the simulated and measured absorbance for the fabricated MOFS MPA at two polarizations under normal incidence. In this figure, the simulated and measured absorbance follow the similar trend; the measured results revealed higher absorbance below 250 THz and similar absorbance beyond the same frequency for x-polarization and higher absorbance below 160 THz and similar absorbance beyond the same frequency for y-polarization compared to the simulated one. The difference between the simulated and measured results might originate from that although we use VBA to construct a stochastically distributed MOFS MPA, yet, in simulation the array is composed of the identical unit cells while the fabricated unit cells within the array differs to each other. Moreover, even the flat sheets show some deviations within a unit cell, for example, the fabricated sheets are more similar to a trapezoid than a rectangular. In addition, we also included the absorbance spectra of both the simulated MOFS and planar MPA. The measured MOFS MPA outweighs the two in terms of bandwidth and intensity. Here, the MOFS MPA possesses superior absorbance in terms of bandwidth and allowed incident angle and could be roughly predicted by our proposed simulation with VBA. In short, our proposed MOFS MPA revealed fractional bandwidth of 126% at x-polarization with 79.4% average absorbance. The incidence angle could up to 70°. Meanwhile, the MPA also showed fractional bandwidth of 131% with average absorbance of 71.61% at y-polarization. The incidence angle is also up to 70° with the similar absorbance performance. Compared to other works, our proposed MPA possessed advantages such as larger bandwidth compared to fractional bandwidth of 62% in Ref.^32^ (the average absorbance is 84.6%), incident-angle independence compared to the acceptable incidence angle of 60° and 40° for TM and TE modes in Ref.^28^ (the fractional bandwidth and average absorbance are 147% and 81%, respectively), and easy fabrication method compared to multiple lithography and alignment procedure in Ref.^29^ (the fractional bandwidth and average absorbance are 150% and 72.2%). Still, it is worth mentioning that lossy metal such as vanadium, tungsten and titanium was employed in Refs.^28,29^, thus resulting in better absorption efficiency^39,40^. We believe that if we replace aluminum into such lossy metal, we could further boost the absorption efficiency, bandwidth, and incidence angle independence of our proposed MOFS MPA.

## Conclusions

With the abovementioned simulation and measurement, we designed, fabricated and characterized the multiple oblique-flat-sheet based metamaterial perfect absorber. First of all, the SOFS MPA was simulated to validate that an oblique flat sheet could provide multiple optical paths to enable multiple absorption peaks; furthermore, when conducting oblique angle deposition, multiple oblique-flat-sheets were developed and to simulate this sample, we employed VBA to construct many oblique flat sheets with various dimensions. The simulated results show better absorbance and wider incident angles than the planar MPA, corroborating that by simply adjusting the deposition angle, we could achieve much better absorption performance without difficult fabrication process. In measurement, the simulated and measured results under normal incidence are similar to each other with the same tendency, suggesting that in the future, we could roughly predict the result in the aids of simulation. Again, the measured absorbance outweighs the ones of the planar MPA. To conclude, we provide, in this work, a simple method to achieving a broad bandwidth and incident angle MOFS MPA that would be a potential candidate in the fields of energy harvesting, sensing and imaging and stealthy technology.

## Methods

### Construction of stochastically distributed OFS MPA

To construct a stochastically distributed OFS MPA, we analyze SEM images of the as-fabricated sample to roughly determine ranges of each controlled parameter. Here, we consider amounts, lengths, widths and their distribution to better approach to the fabricated sample. First, the total amount of oblique flat sheets was randomly chosen from 15, 20 and 25. Then, the length and width of each oblique flat sheet was determined based on two maximum values that were multiplied by a randomly proportional factor (less than 1), for example, the maximum length and width in our simulation were 740 nm and 200 nm with a proportion factor ranging from 0.2 to 0.8. On the other hand, the distributions of the oblique flat sheets also deviate a lot; yet, we should still avoid potential aggregation of the adjacent sheets. To achieve this target, we divided the length of a seed layer in the y direction into three pixels while in the x direction, it is segmented into the total number of flat sheets divided by 3. Through coupling between the segmentation and OFS distributions, we could avoid denser distribution within a specific regime. Note that once an oblique flat sheet is created, the next sheet would be created at the next segment. In addition, from SEM, we could observe there appear some shorter OFSs. To reflect such structures, we set up a total 100 shorter OFSs with a maximum length of 50 nm and a proportional factor of 0.1–0.5 and a maximum width of 25 nm and a proportional factor of 0.1–0.8. The interval of the proportional factor is 10%. Through this construction process, finally, we obtained oblique-flat-sheet metamaterial perfect absorber with S-parameters that are similar to the measured results.
